# Evaluation of oxygen prescription in relation to hospital admission rate in patients with chronic obstructive pulmonary disease

**DOI:** 10.1186/1471-2466-14-127

**Published:** 2014-08-05

**Authors:** Alice M Turner, Sourav Sen, Cathryn Steeley, Yasmin Khan, Pamela Sweeney, Yvonne Richards, Rahul Mukherjee

**Affiliations:** 1 College of Medical and Dental Sciences, University of Birmingham, Birmingham B15 2WB, UK; 2 Department of Respiratory Medicine & Physiology, Heart of England NHS Foundation Trust, Birmingham Heartlands Hospital, Bordesley Green, Birmingham B9 5SS, UK; 3 Birmingham East and North (BEN) PCT, Fourth Floor, Waterlinks House, Aston, Birmingham B7 4AA, UK

## Abstract

**Background:**

Long term oxygen therapy (LTOT) has a strong evidence base in COPD patients with respiratory failure, but prescribing practices are recognized to need reform to ensure appropriate use and minimize costs. In the UK, since February 2006, all Home Oxygen prescription is issued by hospitals, making respiratory specialists totally in charge of home oxygen prescription. It has been widely noted that inappropriate home oxygen, often for intermittent use (“short burst”), is frequently prescribed in patients with COPD and related conditions with the intention to prevent hospital admissions outside of evidence based LTOT guidelines. We participated in a national Lung Improvement Project aimed at making LTOT use more evidence based. We utilised this unique opportunity of studying the effect of removal of oxygen from COPD patients (who did not meet LTOT criteria) on hospital admission rates.

**Methods:**

Primary and secondary care data sources were used to identify patients with COPD in a single primary care trust who were admitted to hospital at least once due to COPD between April 2007 and November 2010. Admission rates were compared between LTOT users and non-users, adjusted for age and COPD severity. LTOT users were further studied for predictors of admission in those appropriately or inappropriately given oxygen according to NICE guidance, and for admissions before and after oxygen receipt, adjusting further for co-morbidity. Mortality and economic analyses were also conducted.

**Results:**

Readmission was more likely in LTOT users (3.18 v 1.67 per patient, p < 0.001) after adjustment for FEV1 and age by multiple regression. When stratifying by appropriateness of LTOT prescription, adjusting also for Charlson index and other covariates, FEV1 predicted admission in appropriate users but there were no predictors in inappropriate users. In longitudinal analyses admission rates did not differ either side of oxygen prescription in appropriate or inappropriate LTOT users. Specialist assessment resulted in cost savings due to reduced use of oxygen.

**Conclusions:**

Admission to hospital is more likely in LTOT users, independent of COPD severity. Oxygen use outside NICE guidance does not appear to prevent admissions.

## Background

Oxygen has a strong evidence base for use in respiratory failure in chronic obstructive pulmonary disease (COPD), following the landmark long term oxygen therapy (LTOT) studies in the 1980s [1-3]. These studies underpin the recommendation [4] that oxygen should be used for at least 15 hours per day in patients with COPD who have a pO2 when well < = 7.3 kPa, or <8.0 kPa if polycythaemia, pulmonary hypertension or cor pulmonale are present. In patients with less severe hypoxaemia trials are ongoing to establish whether oxygen supplementation has a role [5]. The Royal College of Physicians [6] and the Department of Health [7] have extensively reviewed the way in which services are provided for oxygen use in COPD, as it became apparent that 85000 patients in the UK receive oxygen (at a cost to the NHS of over £110 m), yet in up to 43% of them it is either unused or unlikely to give clinical benefit [7]. A few cost-effectiveness studies have been conducted in the UK, based on which a number of improvement projects for home oxygen assessment and review (HOS-AR) services have been, and are being, conducted to determine the best methods of reform/rationalisation [8-11].

More recently a review in the USA concluded that LTOT was cost-effective based on quality adjusted life years gained (QALYs) if used according to current evidence [12]. Indeed most of the savings likely to result from new HOS-AR systems are from removal of oxygen which is not clinically indicated; this has been proven in several studies [13,14]. Whilst LTOT prescription should be based on blood gas results, and aims to improve survival [1-3], previous estimates of the economic value of oxygen service reform have not taken effect on admissions into account. COPD admissions are recognized as a large burden to the UK health economy; hence we felt that a detailed consideration of the effect of rationalizing oxygen use on this was warranted.

The primary purpose of this audit/study was to explore the belief relating to emergency hospital admission prevention by inappropriate home oxygen prescription, which motivates some healthcare providers to do so and continue to practise, although little robust data on this is available. This audit/study was possible due to our hospital’s participation in a national Lung Improvement Project, aimed at making LTOT use more evidence based.

In this study we also aimed to assess the scale of the problem of inappropriate LTOT prescription in COPD, the relationship of LTOT prescription to admissions, and to model the health economics of oxygen savings when taking admissions into account.

## Methods

### Patient and admission data

Data from the Secondary Uses Service (SUS) for hospital spells involving patients from the Birmingham East and North (BEN) PCT admitted as an emergency and classified with a Healthcare Resource Group (HRG) code relating to COPD was identified by an information manager within the PCT for the period 1st April 2007 to 30th November 2010. Concurrent data from Air Products, the provider of home oxygen supplies to BEN patients, was used to identify oxygen users; charging data enabled identification of those in receipt of LTOT. Relevant emergency spells from the SUS dataset including length of stay (LOS) for each spell were recorded for patients and classified according to receipt of LTOT status (ever v never) along with the start date of the service and, if relevant, the date at which LTOT was withdrawn. A tag identified whether each spell was before, during or after the provision of LTOT. Both the SUS and Air Products database use NHS number to identify patients, enabling date of birth and FEV1 measurements to be gathered from primary and secondary care records.

For LTOT users secondary care records from the oxygen assessing service provider (Heart of England Foundation NHS Trust; HEFT) were reviewed to determine if the patient had ever seen a respiratory specialist and undergone oxygen needs assessment. Sufficient data to determine oxygen needs was defined as a definite diagnosis of COPD (post bronchodilator FEV1/FVC <0.7), with oxygen saturations, and ABG if saturations were < =92% when stable. Appropriate oxygen prescription was defined as that listed in NICE guidance [4]. Co-morbidity was recorded and classified using the Charlson index, chosen as a valid method to quantify co-morbid disease [15] and good predictor of admission [16]. Mortality and cause of death were taken from hospital bereavement records, with cross checking against PCT records. Patients were excluded from further analysis if the data suggested they did not have COPD, or there were inconsistencies bringing data quality into question.

The study was approved by the local Lung Improvement Project Board as a retrospective audit registered with the NHS. As it was a service evaluation by members of the clinical team, it did not require ethics approval as authorized by the Lung Improvement Programme Board constituted by National Health Service (NHS) Trusts and conducted by its employees. No identifiable data was viewed by anyone outside the oxygen prescribing team who clinically managed the patients, in keeping with the principles of the Data Protection Act 1998.

### Statistical analysis

All analyses were carried out in SPSS (version 16.0). Mean number of admissions was compared between LTOT users and non-users with COPD using the t-test, and then after regression, adjusting for age and FEV1 (% predicted), used as continuous variables. In the HEFT dataset all univariate comparisons of appropriate v inappropriate oxygen prescription were carried out using the t test or Mann Whitney, for parametric and non-parametric data respectively, or the Chi square test for frequency variables. Multivariate analysis assessing associations of admission rate examined age, FEV1, gender and Charlson index as predictors, and was carried out separately for appropriate and inappropriate LTOT users. Missing data was dealt with on a case basis, seeking it from additional data sources if possible. Finally paired tests were used to compare admission rate/year either side of oxygen receipt for patients in whom this data was available.

### Economic modeling

Cost/day of LTOT was taken from typical charging data, and cost for an average COPD admission from current NHS tariffs.

## Results

### All COPD patients

There were 1942 patients identified with COPD, of whom 295 had received oxygen during the study period (Table 1). The raw data suggested that oxygen prescription as LTOT on discharge from hospital strongly predicted subsequent admission (3.18 v 1.67 readmissions/patient, p = 1.8 × 10^-12^). Post bronchodilator spirometry was available on 1047 (54%) of patients; a further 22% had their COPD diagnosed by a respiratory consultant, but raw spirometry data was not available. Characteristics of the cohort with spirometry available, such that we are confident of the diagnosis of COPD, are shown in Table 2. Multiple regression analysis in this subgroup seeking predictors of number of admissions showed that neither age (p = 0.34), nor FEV1 (% predicted) were significantly associated (p = 0.11). However LTOT remained significantly associated, with an odds ratio of admission of 1.53 (95% CI 1.09-1.97, p < 1.0 × 10^-6^) in LTOT patients. A similar relationship was seen to total length of stay, with LTOT patients being much more likely to have an LOS more than 6 days (OR 4.5; 95% CI 3.1-6.7, p < 1.0 × 10^-6^). This emphasizes the importance of assessing for LTOT during stability and not using it for the sake of pure “admission avoidance” outside of the guidelines.

**Table 1:** Hospital spells for COPD patients stratified by LTOT status (includes all patients classed as COPD in hospital episode statistics/SUS Data)

**Hospital spells**	**Never LTOT n = 1647**	**Received LTOT n = 295**
1	179	117
2-5	415	137
6-10	40	31
11-20	11	8
21+	2	2

**Table 2:** Characteristics of subset of patients with COPD diagnosis confirmed by spirometry

	**Non-LTOT n = 706**	**LTOT n = 189**	**p**
Number of admissions	1 (1–2)	2 (1–4)	<0.0001
Total length of stay (days)	5 (2–12)	17 (7–34)	<0.0001
FEV1 (litres)	1.14 (0.78-1.58)	0.73 (0.56-1.04)	<0.0001
FEV1 (% predicted)	51 (37–70)	35 (27–50)	<0.0001
Age (years)	73.95 (65.47-81.00)	72.82 (0.72)	0.759

Data is shown as median (IQR) as all were non-normally distributed. Age was normally distributed in the LTOT group and is therefore shown as mean (SE). This was also the only feature that did not differ between LTOT and non-LTOT groups.

### COPD patients given oxygen

There were 295 patients in this group, 20 were excluded from further analysis because records showed oxygen was removed before it was commenced, or that the patient had died before commencement, thus invalidating their record. 263 of the patients prescribed oxygen had seen a respiratory physician and spirometry was carried out in 201 patients within a year of oxygen prescription. 124 had a detailed oxygen assessment including arterial blood gas (ABG) sampling. In 38 cases oxygen could be stopped after this assessment; a further 20 had oxygen stopped on the basis of saturations and clinical review alone, such that ABG was not required. This equates to a cessation rate of 40.3% on specialist review. An additional 16 patients had a palliative oxygen prescription (died within a month of commencement, with documented terminal disease, either end stage COPD or co-existent malignancy). In all 12 patients who had not seen a respiratory physician there was insufficient information to determine if oxygen was required, suggesting a strong trend to more robust, evidence-based prescription if seen by a specialist (p = 0.066). In patients where there was sufficient data to assess oxygen needs, and it was not given for palliation purposes there were no differences in age, gender, lung function or co-morbidity (Charlson index) between appropriate and inappropriate prescriptions (all p > 0.15). Patient characteristics are shown in Table 3.

**Table 3:** Characteristics of patients prescribed oxygen

Age (years)	73.03 (3.13)
Male gender	119 (43.30)
FEV1 (litres)	0.85 (0.56-1.00)
FEV1 (% predicted)	47.18 (8.08)
FEV1/FVC	0.49 (0.05)
Charlson index	3.00 (0.58)
pO2 (kPa) on air	6.60 (6.20-6.73)
pCO2 (kPa) on air	5.89 (0.48)
Days received oxygen	190 (40–1289)
Time to death from oxygen being started (days)	827.63 (165.26)
Time between death and oxygen cessation (days)	
Oxygen stopped before death	324.50 (161.00-603.75)
Oxygen stopped after death	8.50 (5.00-17.00)

The table shows the characteristics of those patients that received oxygen. Normally distributed data is shown as mean (SEM) and non-normally distributed data as median (IQR), whilst the single frequency variable is highlighted in bold type and is shown as n (%).

#### Mortality

64% (n = 176) of patients who received oxygen died during the follow up period; of these 47% (n = 84) had a purely respiratory cause of death and 9.1% (n = 16) a non-respiratory cause, with the remainder having respiratory disease listed as a contributory factor. Purely respiratory deaths were more likely in those appropriately given oxygen (p = 0.044). On the whole oxygen prescription ceased swiftly after patients died (Table 3), although in one case it continued for 767 days after death.

#### Admissions

Regression analysis assessing predictors of admission in those appropriately given oxygen showed that FEV1 was the only significant predictor of readmission (p = 0.045), whilst no predictors were found in those inappropriately prescribed it. There were no differences in admission frequency or length of hospital stay either before, during or after oxygen prescription, adjusted for the time of each period per patient (all p > 0.54) when comparing those appropriately and inappropriately prescribed oxygen.

There were 48 patients in whom oxygen was started during the study period and data available both before and during oxygen prescription. In these patients admission rate did not differ after being given oxygen (2.90 (3.26) v 2.17 (1.53); p = 0.15), nor did length of stay (p = 0.94). When this group was sub-stratified by whether oxygen prescription was appropriate or not the significance of these figures did not change; in the group given oxygen appropriately mean admission frequency fell by 0.4/year (p = 0.31) and in those given it inappropriately by 2/year (p = 0.29). The rate of admission in the inappropriate oxygen prescription group was very variable, ranging from a gain of 2 to a reduction of 6 admissions. In 17 patients where oxygen had been prescribed inappropriately rates and lengths of stay did not differ after it was removed (both p >0.56). In those patients inappropriately prescribed oxygen no admissions related to harm from oxygen were identified.

### Economic impact of oxygen service

No adverse impact of removal of oxygen from patients given it outside current guidance was found on hospital admissions nor were there any significant benefits on hospital admission rates from prescribing oxygen, regardless of need. Consequently we anticipate no hidden economic impact of reform of oxygen services on secondary care usage by COPD patients in the UK. Cessation in the 40.3% of LTOT patients where prescription was inappropriate would save £71000 in a population of 300000 (equivalent to this PCT), and £30 million in the UK each year, allowing for the slight increase in ambulatory oxygen use. Oxygen cessation on registration of death could save up to a further 10% per annum in unreformed services, based on the average days given after death.

## Discussion

Our data suggests that home oxygen prescription outside of evidence based guidelines does not reduce admission rates and confirms that robust oxygen assessment systems can benefit the local health economy. This analysis holds true in a large group with limited phenotypic data, and in a highly characterized dataset, and even in individual patients assessed longitudinally either side of oxygen prescription. This 3 level analysis is a significant strength of our study. Many studies have attempted to define predictors of exacerbations, and hospitalizations in COPD. The ECLIPSE study identified severity of COPD, presence of gastro-oesophageal reflux disease, poor quality of life score and white cell count to independently predict exacerbation rate, but did not report any effect of oxygen use [17]. Admission for exacerbation is more common in severe COPD [17] and in oxygen users [18], whilst readmission relates to BODE index [19] and psychosocial factors [20]. Since arterial oxygen levels are predicted by lung function [21], and the BODE index has FEV1 as one of its major components [22] taken together this data suggests that we should expect readmission to hospital more frequently in LTOT users due to their more severe lung disease. Our initial analysis appeared to confirm this, in that oxygen users throughout the PCT catchment area came into hospital more often, and their lung function was worse (Table 2). However if disease severity were the main predictor of both respiratory failure and readmission we would have expected this difference to reduce more markedly when adjusted for FEV1. Since this was not the case, the data suggested that receipt of oxygen was adversely impacting admissions independent of COPD severity. This is in direct contrast to the perception amongst healthcare providers that it may reduce admissions, and therefore warranted further exploration. The PCT level data did not allow us to assess appropriateness of oxygen prescription or co-morbidity in relation to admissions, such that a more detailed consideration of secondary care records for those patients who had received oxygen was carried out.

The secondary care data allowed us to categorise patients according to their oxygen needs, as defined by current guidance [4], and adjust for significant co-morbidity by use of the Charlson index. We were also able to look at admission rates longitudinally, according to oxygen receipt status in those patients where oxygen was commenced or removed during the data collection period. This makes the analysis much more robust than the PCT level results. The data showed that admissions were no more common in patients appropriately given oxygen, compared to those where it was given inappropriately. Whilst COPD severity (FEV1) was a good predictor of admission in patients where LTOT was indicated, neither this, age or co-morbidity associated with admission in those inappropriately given oxygen. Whilst we can only speculate on unmeasured factors that could be driving hospital attendance, it is of note that psychosocial issues were not accounted for. Furthermore, in the longitudinal analyses no evidence of a change in hospital admission rates was seen when receiving oxygen, regardless of whether this was appropriate. This suggests that prescribing oxygen as a bridge to LTOT assessment in borderline patients on discharge is not likely to be of benefit, and concurs with other studies of moderately hypoxaemic COPD patients showing that receipt of oxygen does not reduce hospitalisations [23].

We also chose to look at mortality in the patients given oxygen, as it was apparent from the high Charlson indices, poor lung function and ABG results that this population was very unwell, and reducing mortality is a key aim of LTOT use. We confirmed that respiratory deaths were common, highly likely to occur within a few years of starting oxygen, and more likely when oxygen is indicated. It is important to note that we found no evidence of harm resulting from oxygen prescription, particularly there were no admissions or deaths related to oxygen use in patients given it against current guidance.

In our study LTOT was stopped in 40.3% of patients after specialist review, a figure consistent with previous work [24], suggesting our results are likely to be generalisable. It is possible that some of these patients may require oxygen other than LTOT, and therefore assessment for ambulatory oxygen alone – this was not a feature we could examine in our data, although it has been suggested that such assessments may be a drain on oxygen assessment services [24]. There are significant numbers of COPD patients who exhibit desaturation on exercise, although they are normoxaemic at rest. This predicts mortality [25] and subsequent need of LTOT [26]. Trials of ambulatory oxygen have varied in their design and outcome measures, such that a recent review concluded further evidence was needed [5]. Cochrane continue to advocate individual assessment [27], rather than a guideline based approach, for this reason. It remains unclear whether home ambulatory oxygen use is beneficial; self-reported improvements in activity levels and exercise tolerance did not match well to objective measures of exercise capacity in previous work [28]. Short burst oxygen therapy (SBOT) has been the subject of a systematic review and meta-analysis, and shown to provide no symptomatic benefits [29]. Consequently use of home oxygen outside of the setting of LTOT remains controversial [4]; when the Long term Oxygen Treatment Trial (LOTT; http://clinicaltrials.gov/show/NCT00692198) reports results, some of these questions may be answered. Given this uncertainty, whilst we acknowledge that lack of data on ambulatory oxygen needs is a potential weakness we do not think it clinically significant.

The further important limitations of our study are: (a) due to the nature of the pre-2012 contracts with home oxygen providers in England, we do not have concordance data on actual oxygen use therefore the hospital admission rates amongst COPD patients prescribed LTOT could not be corrected to actual oxygen use; (b) No data was available for current smoking habits of LTOT users; (c).

Since there was no effect of oxygen (prescribed outside evidence based LTOT guidelines) on emergency hospital admission rates in our study, the economic benefits of an oxygen assessment service broadly confirm the Department of Health findings [7] that savings can be made through multidisciplinary LTOT assessment services. Locally our oxygen service does have any ringfenced budget for any extra secondary care staff, thus we have not accounted for staff costs in our analyses, although we recognize that these could reduce the impact of the savings. Nevertheless our finding that oxygen does not adversely impact admissions is of value, and the potential for harm from inappropriate oxygen – although not shown herein – should further strengthen the support to introduce HOS-ARs across the UK. The quality of assessment when seen by a specialist was higher, as indicated by the fact that insufficient data to guide oxygen prescription was available in patients given it by a generalist, again concurring with guidance that experienced practitioners should manage oxygen prescriptions.

## Conclusions

We conclude that robust oxygen assessment practices in COPD patients are valuable to the health economy, by direct savings, with no hidden costs or adverse events related to admissions to hospital.

## Nomenclature

Secondary Uses Service (SUS), The Secondary Uses Service (SUS) is primarily a data warehouse that provides access to anonymous patient-based data for purposes other than direct clinical care such as healthcare planning.

PCT, Primary Care Trusts were largely administrative bodies, responsible for commissioning primary, community and secondary health services from providers, now replaced by clinical commissioning groups in the UK.

Healthcare Resource Group (HRG) code, The HRG system is used by Payment by Results, an activity based payment system rolled out in the NHS in England since 2004, the code comprising patient events that have been judged to consume a similar level of resource.

Emergency spells/hospital spell, An emergency spell relates to the whole hospital stay of a patient, from admission to discharge. For complex patients the Spell may contain many episodes of care under different consultants.

## Competing interests

The authors have no competing interests to declare in relation to the contents of the manuscript.

## Authors’ contributions

AMT collected data, conducted data analysis and wrote the manuscript; SS and CS collected data; YK, PS and YR created data collection sheets, set up database and collected data; CS also performed data analysis. RM conceived the study supervised the work and reviewed the manuscript – he is the guarantor of the paper. All authors had access to the whole dataset for analysis if required. All authors read and approved the final manuscript.

## Pre-publication history

The pre-publication history for this paper can be accessed here:

http://www.biomedcentral.com/1471-2466/14/127/prepub
